# Compliance of Professional Nurses at Primary Health Care Facilities to the South African Cervical Cancer Screening Guidelines

**DOI:** 10.3390/nursrep11040069

**Published:** 2021-09-22

**Authors:** Nthanyiseni Rangolo, Takalani Grace Tshitangano, Foluke Comfort Olaniyi

**Affiliations:** Department of Public Health, University of Venda, Thohoyandou 0950, South Africa; thendorangolo@gmail.com (N.R.); Takalani.Tshitangano@univen.ac.za (T.G.T.)

**Keywords:** cervical cancer, compliance, primary health care, screening

## Abstract

Despite the availability of the South African cervical cancer screening guidelines at clinics, women still present in district hospitals of Thulamela Municipality with no cervical cancer screening results. Thus, many cervical cancer screenings done at the hospitals often come back positive for cervical cancer at advanced stages. This study was conducted to investigate the compliance of professional nurses at primary health care facilities (PHCs) in Thulamela Municipality to the South African cervical cancer screening guidelines. The study adopted a qualitative approach. Purposive, non-probability sampling method was used to select PHCs and recruit eligible participants. Sample size was determined by data saturation. A digital recorder was used to log individual responses during interview sessions. Data from the digital recordings were transcribed verbatim. Results were analysed and interpreted in accordance with the consolidated criteria for reporting qualitative research (COREQ) checklist. This study established that clinic professional nurses are non-compliant to the South African cervical cancer screening guidelines owing to several challenges they face, such as inadequate knowledge of the cervical cancer screening guidelines, shortage of resources, shortage of staff and patients’ factors. We recommend a strengthening of the South African cervical cancer screening guideline, in-service trainings and workshops on cervical cancer and cervical cancer screening guideline as well as improvement on patients’ education.

## 1. Introduction

Cervical cancer is a type of cancer that occurs in the cells of the cervix. Various strains of the human papillomavirus (HPV) play a role in the aetiology of most cervical cancers. Cervical cancer continues to be a major public health problem globally as it is one of the leading causes of morbidity and mortality amongst the gynaecological cancers and it has been associated with the mortality of one quarter million women annually, with 80% of deaths occurring in developing countries [1,2,3]. It may take up to ten years for high-grade changes in cervical cells to develop into cervical cancer if left untreated; thus, cervical cancer screening tests are done to detect these changes before they advance [4]. Cervical cancer occurs most commonly amongst women who have never been screened or have not been screened within the previous 5 years [5]. It is a deadly disease once it reaches the invasive stages, but it is preventable and treatable if detected at its early stages [3,4,5,6].

Developing countries carry a higher burden of cervical cancer compared with developed countries [7]. Many developing countries are unable to implement a population-based cervical cancer screening programs as a result of poverty, lack of resources and infrastructure [8]. Thus, “the incidence of cervical cancer is steadily increasing in Sub- Saharan Africa with more than 75,000 new cases and close to 50,000 deaths a year, and the WHO has predicted that cervical cancer will kill more than 443,000 women per year worldwide by 2030, with nearly 90% of them in Sub-Saharan Africa” [9]. In South Africa, women diagnosed with cervical cancer were estimated to be 7735 new cases in 2012 and 4248 died from the disease [10].

With access to screening and treatment options, the estimated 5-year net survival from cervical cancer is now between 60 and 70 percent in many high-income countries; for example, the incidence of cervical cancer in the United States of America (USA) has reduced in accordance with effective screening practices where cervical cancer screening has been included as part of a woman’s health check-up [2]. The World Health Organization recommends that pap smears be repeated every 3–5 years for HIV negative women and those whose HIV status is unknown, or every 3 years for HIV positive women [11]. Unfortunately, the uptake of cervical screening services is very low among women, even in countries where the service is free of charge and widely available. This low uptake has been associated with a lack of knowledge about the disease and screening practices [2,3,4,5,6,7,8,9,10,11,12]. In South Africa, cervical cancer screening programs aim to detect women with unsuspected cancer risk by testing asymptomatic women from the age of 25 years or at any age when Human Immunodeficiency Virus (HIV) has been diagnosed [13,14]. The test is expected to be conducted by nurses at the primary health care facilities during their first consultation with eligible women. The South African cervical cancer screening guideline (2017) adopts the WHO screening intervals and recommends that pap smear screening should end at age 55 or hysterectomy only after previous negative tests, and should never end in HIV positive women [15]. Despite the availability of the South African cervical cancer screening guidelines, women are still seen in the outpatient departments of rural district hospitals and referred from primary health care facilities with no cervical cancer screening results. Thus, the screenings are done at the hospital and results often come back positive for cervical cancer. Such practises pose a risk of delayed cervical cancer diagnoses and its discovery at an advanced stage, leading to increasing cervical cancer mortality rates. This study was therefore conducted to describe the compliance of clinic professional nurses to South African cervical cancer screening guidelines, to explore challenges faced by professional nurses regarding cervical cancer screening practices and to describe the perceived strategies of improving compliance to the South African cervical cancer screening guidelines.

The study was conceptualized by the first author of this article, a professional nurse who at the time of the study was working in the oncology unit of a district hospital in Thulamela Municipality and had observed with disappointment how women who had never been screened for cervical cancer present at the hospital where she worked with advanced stages of cervical cancer. She had some trainings on cervical cancer screening as a part of the South African nursing training curriculum, thus, she expected that other nurses with the same training would be able to conduct the screening tests and discover cervical abnormalities at the early stages. Her experience at the district hospital prompted her to conduct this study, to gain insight into the reasons why the professional nurses at the clinics were not conducting the screening tests. This study was reported in adherence with the consolidated criteria for reporting qualitative research (COREQ) guidelines as developed by Tong, Sainsbury and Craig [16].

## 2. Methods

### 2.1. Study Design

This study adopted a qualitative approach, which facilitated easy exploration of professional nurses to understand, interpret and describe the compliance of clinic professional nurses to the South African cervical cancer screening guidelines in Vhembe District, Limpopo Province.

### 2.2. Population

All Professional nurses working at Primary health care facilities under William Eddie and Mutale local areas of Thulamela Municipality.

### 2.3. Sampling

A purposive, non-probability sampling technique was used to select clinics and eligible participants who possess the relevant information required to answer the research question of this study.

### 2.4. Inclusion Criteria

i. Professional nurses who have a minimum of 6 months’ experience at any primary health care facility.

ii. Professional nurses who screen patients in consultation rooms.

### 2.5. Sample Size

The sample size of the study comprised of 2 professional nurses per clinic in each selected local area. The researcher earmarked to interview a total of 42 professional nurses, 24 from Mutale local area and 18 from William Eddie local area. During recruitment of participants, only 30 professional nurses volunteered where 20 were from the Mutale local area and 10 were from the William Eddie local area. Data saturation was reached at participant number 18 in the Mutale local area and number 7 in the William Eddie.

### 2.6. Ethical Considerations

Ethical clearance was obtained from the University of Venda and Research Ethics Committee (SHS/19/PH/15/1406). Permission to conduct the study was also obtained from the Department of Health, Limpopo Province (LP–201910–005) and the Department of Health Vhembe district. Written informed consent was obtained from each participant and other ethical principles were duly followed in the course of the conduct of the study.

### 2.7. Data Collection Tool

An unstructured interview guide was developed with three questions as follows: 1. At this clinic, how do you screen any female patient who enters your consultation rooms? 2. What are the challenges you face when you try to comply with the South African cervical cancer screening guidelines? 3. What do you think must be done in order for you to comply with the South African cervical cancer screening guidelines? Follow up and probing questions were asked after the participants’ responses. English language was used as the means of communication. Participants were not requested to provide their names during the interview; however, pseudonyms were assigned to them for the purpose of reporting the study. The tool was pre-tested at Shayandima clinic, another clinic in Thulamela municipality, which did not form a part of the main study.

### 2.8. Measures to Ensure Trustworthiness

Methods that were adopted by the researcher to ensure credibility of the study included establishment of rapport, which was achieved when the researcher outlined the necessary information regarding the research after the participants gave consent. Credibility was achieved by showing the participants the field notes and allowing them to listen to the recorded interviews through the voice recorder during data collection to confirm if they represent their opinions accurately. Also, after the interviews were transcribed, the transcription was made available to the participants, for them to review and verify their statements. Dependability was achieved by describing the research findings, interpretations and recommendations, using an auditable trail so as to corroborate that the investigation was supported by data and was internally coherent.

### 2.9. Data Collection Process

Data was collected by the first author, who visited the selected PHC facilities to introduce the topic of interest, explain the rationale, purpose, benefits and methodology of the study to professional nurses and requested their participation in the study afterwards. She had no prior relationships with any of the participants and they in turn knew nothing about her beyond the information she provided for them in relation with the research. Those who volunteered to participate in the study were recruited and appointments were made with them for a revisit at times convenient for them for the interviews. Though they were informed of their rights to withdraw from the study at any time they so wished, none of those who volunteered to participate dropped out of the study. On the appointed dates of the interviews, participants were made to sign informed consent forms before the interviews were conducted. Face-to-face interviews were conducted at the professional nurses’ clinic rooms during lunch breaks to avoid interfering with the nurses’ official duties. The participants were interviewed individually and privately, to ensure that the presence of a third party does not affect the participant’s responses. A voice recorder was used to record the interviews and field notes were taken during the interview. Each interview session lasted 30–45 min.

### 2.10. Data Analysis

A thematic analytical approach was used to analyse the data. This approach involves familiarising oneself with the depth and breadth of the data content, generation of initial codes, searching for themes, reviewing themes, defining and naming themes and producing the report [17]. The researcher familiarised herself with the data content by listening to the recorded interviews many times; transcribing the recordings verbatim and reading the transcribed document over and over again, until she was able to generate the recurring themes and sub-themes, which are reported in this article.

## 3. Results

The analysis of the data yielded two main themes, which were further divided into sub-themes. The results are summarized in Table 1.

### 3.1. Theme 1: Professional Nurses’ Screening Practices of Female Patients in Consultation Rooms

#### 3.1.1. Sub-Theme 1: Professional Nurses’ Compliance with Screening Practices

According to the participants, newly diagnosed HIV positive women are screened immediately with the use of a pap smear test and the test is repeated annually during visits to the clinic. The following are excerpts from participants:

*‘All HIV positive female patients who enter my consultation room have Pap smear samples taken annually as part of the HIV management program’ (Maanda).*

*‘It is always a reminder for me to do a pap smear when consulting a HIV positive female’ (Nancy).*

#### 3.1.2. Sub-Theme 2: Professional Nurses’ Non-Compliance Practices

None of the participants of this study has screened asymptomatic women in all the clinics visited. Participants only screen patients as per gynaecological complaints (e.g., lower abdominal pains, vaginal bleeding).

*‘I always remember to do Pap smear when a patient complains of symptoms like lower abdominal pains, and vaginal bleedings (post-menopausal women)’ (Nancy).*

*‘Women coming for family planning and those with heavy vaginal discharges, are a red flag for me to do pap smears (Thendo).*

*‘I really do not see any need of doing pap smear on a patient who has no complaints’ (Rose).*

*‘I only perform pap smears on young women 25 years of age and below at 6 weeks’ post-natal care as per maternity registers recommended check-ups’ (Phyllis).*

*‘Girls are becoming mothers at a very young age and that’s where I perform pap smears as post-natal check-ups’ (Freddy).*

*‘I only do pap smear as per the pap smear screening register which guides us to start at 30 years of age’ (Mashudu).*

### 3.2. Theme 2: Challenges Faced by Professional Nurses Regarding Cervical Cancer Screening Guidelines

The analysis of the data yielded three sub-themes of findings, namely: patient related challenges, such as fear of pain, age, cultural beliefs and lack of knowledge; health care practitioners’ related challenges, such as inadequate knowledge on cervical cancer; and health care system related challenges, such as shortage of staff, inadequate privacy in consultation rooms and shortage of instruments to perform pap smear tests.

#### 3.2.1. Sub-Theme 1: Patient Related Challenges

This sub-theme yielded factors, such as fear, age, cultural beliefs and lack of knowledge.

Fear: The patient’s fears are related to the instruments or the fear of being tagged sexually promiscuous.

*‘After convincing the patient to do pap smear, I showed her all my instrument and her face changes as she said ‘no, no, no that big thing will tear me apart’, then she refused (Conny).*

*‘The patient said, “sister there is no way you will put that big thing of yours inside of me” when I showed her a vaginal speculum’ (Mashudu).*

*‘Some patients request to come for pap smear on a different day as they fear that their friends may assume that they are unfaithful and have more sexual partners, so they opt to do pap smear when they come to the clinic alone (Khathutshelo).*

Age: Older women seem to associate pap smear with active sexual activities, and thus decline the test.

*‘Old women get angry when you explain about pap smear, some asked if she looks like a prostitute at her age, if that is why I think she must do a pap smear’ (Hlamarisani).*

*‘An old woman once said, “my child, I am too old to be sexually active and so why should I do a pap smear?”’ (Maria).*

Cultural beliefs: In the culture of the people where this study was conducted, younger women are not supposed to see the nakedness of older women. This affects the older women’s consent for screening.

*‘It is culturally considered a taboo to see an old woman naked, therefore, patients will ask for older nurses to do pap smear instead of young nurses, and that means she will not have pap smear as we have no older nurses that she prefers’ (Thendo).*

Lack of knowledge: Many patients do not have sufficient knowledge about the predisposing factors of cervical cancer.

*‘Unlike HIV/AIDS, cervical cancer is a disease that most patients know nothing about, so when I explain predisposing factors (like early sexual encounters and multiple sexual partners), it makes patients not want to do pap smears but others find the information helpful and therefore agree to have the test’ (Norah).*

#### 3.2.2. Sub-Theme 2: Health Care Practitioners’ Related Challenges

Inadequate knowledge of cervical cancer: Most of the participants do not have adequate knowledge about cervical cancer, and thus find it difficult to explain it to patients.

*‘I don’t know much about cervical cancer and hence I avoid explaining something I am not sure of to the patients; I always overlook the pap smear talk due to that’ (Oluga).*

*‘Since I came to this clinic, I never received any training on cervical cancer so I am not sure enough to tell any person about it, let alone a patient’ (Nancy).*

*‘I’ve only attended one training on cervical cancer but I am not yet confident about it’ (Maano).*

Lack of skills to perform a Pap smear: Many of the participants in this study do not have the necessary skills to perform a pap smear.

*‘I did Pap smear five times since I worked in the clinic, my results always come back insufficient (to be repeated), so we need workshops’ (Maria).*

*‘I am not sure what to look for in a pap smear’ (Tshedza).*

*‘I have always taken smear inside the vaginal wall as how I was shown by my senior in this clinic’ (Amanda).*

Attitudes of Nurses: Many participants do not have a good attitude towards cervical cancer screening.

*‘Pap smear takes long to complete and there’s always a long queue of people waiting to be seen, and besides I’m in no mood to always look at other women’s private parts’ (Tshililo).*

*‘As a male nurse, I leave the pap smears to the female nurses, I don’t want to be sued for sexual harassment’ (Gudson).*

*‘If a patient refused to be done Pap smear, I do not have time (she frowns and shakes her head) to beg or convince her as there is always a long queue waiting to be assisted’ (Ntondeni)*

Ignorance: Some Nurses are not aware about the existence of cervical screening guidelines.

*‘I really have no idea that there is a guideline for cervical cancer screening’ (Elekanyani).*

*‘Since I qualified 5 years ago, I’ve heard about cervical cancer screening but has never done even one Pap smear, I can’t say I know how to do it though’ (Tshimangadzo).*

#### 3.2.3. Sub-Theme 3: Health Care System Related Challenges

Shortage of staff: Many participants complain bitterly about shortage of staff in the clinics.

*‘Look at the queue outside, do you think 2 professional nurses will manage if performing pap smears in-between so many patients? As it is, we are not managing to achieve the expected waiting time for each patient in this clinic, so if we add pap smears we will have problems’ (Tshedza).*

*The workload is too much already and we are not managing being 3 professional nurses per shift’ (Joyce).*

Inadequate privacy in consultation rooms: Participants are unable to perform pap smears sometimes, because of a lack of adequate privacy of patient.

*‘Anyone can just come into the room while I am busy with a pap smear, this is invading the privacy of the patient, look at the door, it cannot lock’ (Maimo).*

Shortage of instruments to perform Pap smears: Some participants complained of shortage of pap smear instrument or incapacitation to sterilize the instruments.

*‘Vaginal speculums are only 5 in this clinic’ (Tshilidzi).*

*‘The autoclaving machine is not working for the past 4 months, so how am I supposed to do pap smears?’ (Norah).*

*‘After convincing the patient to do pap smear, I found out that there are no slides to use, so I have lost a patient then’ (Zwonaka).*

## 4. Discussion

This study showed that professional nurses who are practicing in the clinics located in the William Eddie and Mutale local areas of Thulamela Municipality do not comply fully with the cervical screening guidelines of South Africa, in terms of screening all asymptomatic women from the age of 25 years, though they are compliant with the aspect of screening women who tested positive to HIV. Also, the cervical cancer screening registers consulted in all the clinics have 30 years, rather than 25 years as the age of initiation of screening. Women less than 30 years who are screened because they tested positive to HIV are not included in the monthly statistics, thus it becomes difficult to estimate accurately the number of women from the recommended age of 25 who have been screened for cervical cancer. This observation is supported by Botha and Dreyer who observed that women are being screened from the age of 30 years in South African public health care facilities according to the national cervical cancer prevention programme at the time of the publication of the guidelines in 2017 [15]. This study was conducted in 2019 and it shows that the new recommendation has not been implemented. We recommend that the new age of initiation of cervical screening be implemented in all health care facilities across the country, to facilitate early detection of cervical cancer and prompt treatment where necessary.

The reports of fear of the pap smear screening test due to expectation of pain or “tearing apart” of the private parts as reasons why some patients refuse to be screened showed a lack of proper understanding of the procedure on the part of the patients. This can also be related to a low perception of their susceptibility to cervical cancer. This perception has been associated with screening behaviours [18,19]. Health care workers at the clinics should educate females, especially young females on the risk factors for cervical cancer, the importance of the screening test and as such motivate them to have pap smear tests. Maseko et al. recommend that pap smear campaigns should be done, and that the messages in the campaigns should be organized in such a way that they take into account the educational, sociocultural and religious barriers that are hindering the women to access cervical cancer prevention services [20]. Increased community awareness should emphasise that screening would not involve a pelvic examination, can be done in privacy and would involve providers’ counselling to encourage broader participation in organized screening campaigns [21].

This study also showed that many of the professional nurses who participated have little knowledge about cervical cancer and pap smear and as such have no confidence in performing the screening test. Many of them blamed this on a lack of training. This is in agreement with a prior study which showed that 60.6% of professional nurses working in gynaecological clinics had no prior work experience and receive no in-service training on pap smear [22]. It has been recommended that the training curriculum of nursing and medical schools be reviewed to incorporate practical skills on cervical cancer screening, such that nurses and doctors may graduate with adequate skills to effectively screen patients for cervical cancer [23,24,25]. Furthermore, continuing education for health care workers and in-service trainings of professional nurses will give them the required knowledge and confidence to do pap smears, thus, this should be encouraged in all healthcare facilities. Training of more cervical cancer prevention service providers who should be fairly distributed between urban and rural areas is also recommended, and service providers should be supported through periodical supervision [20].

This study also elicited the negative attitude some professional nurses have towards pap smears. Negative nurse’s attitudes towards cervical cancer screening hinder both the nurse and the patient to do the test as negative attitudes amongst health care practitioners have been shown to affect screening-seeking behaviours from women [20,21,22,23]. The health care related challenges include shortage of staff and instruments. Nurses are overwhelmed with a large number of patients and fewer staff members on duty per time [26]. We therefore recommend employment of more health care workers in the clinics and supply of adequate number of cervical cancer screening equipment in all the clinics.

## 5. Conclusions

The findings revealed that many clinic professional nurses in the study areas have limited knowledge about cervical cancer and pap smear screening with the South African cervical cancer screening guidelines and therefore do not strictly adhere to these guidelines. We recommend a strengthening of in-service trainings and workshops on cervical cancer and cervical cancer screening for clinic professional nurses as well as improvement on patients’ education.

## Figures and Tables

**Table 1:** Themes and sub-themes generated from the participants’ interviews.

Sub-themes	Themes	
	Theme 1: Professional Nurses’ Screening Practices of Female Patients in Consultation Rooms	Theme 2: Challenges Faced by Professional Nurses Regarding Cervical Cancer Screening Guidelines
Sub–Theme 1	Professional nurses’ compliance with screening practices	Patient related challenges
Sub–theme 2	Professional nurses’ non-compliance practices	Health care practitioners’ related challenges
Sub–theme 3		Health care system related challenges

## Data Availability

The data presented in this study are available on request from the first author.
